# Intestinal transit time phenotype is not transferred through gut microbiota transplantation

**DOI:** 10.7717/peerj.21064

**Published:** 2026-05-08

**Authors:** Anna Pii Hjørne, Chiara H. Moretti, Thomas U. Greiner, Nicola Procházková, Henrik M. Roager, Martin Steen Mortensen, Fredrik Bäckhed, Tine Rask Licht, Martin F. Laursen

**Affiliations:** 1 National Food Institute, Technical University of Denmark, Kongens Lyngby, Denmark; 2 Novo Nordisk Foundation Microbiome Health Initiative, Technical University of Denmark, Kongens Lyngby, Denmark; 3 Department of Molecular and Clinical Medicine, University of Gothenburg, Gothenburg, Sweden; 4 Department of Nutrition, Exercise and Sports, University of Copenhagen, Frederiksberg, Denmark; 5 Novonesis, Hørsholm, Denmark; 6 Department of Clinical Physiology, Sahlgrenska University Hospital, Gothenburg, Sweden

**Keywords:** Intestinal transit time, Gut microbiota, Fecal microbiota transplantation, Animal model, Loperamide

## Abstract

Intestinal transit time (TT) varies considerably between healthy individuals and affects gut microbiota composition and activity. Whether differences in the gut microbiota composition also affect the TT is not well elucidated. In this study, we conducted two animal experiments to explore causality between the gut microbiota and TT. In the first experiment, we transplanted two groups of female germ-free (GF) Swiss-Webster mice with fecal material from two healthy human donors with fast and slow TT phenotypes. Following transplantation with human feces, we observed a decrease in TT for both groups of GF recipient mice (from 300 min to 167 min, 95% CI: ±45; and from 369 *vs* 205 min, 95% CI: ±52) corresponding to reductions of approximately 45% in each group, supporting previous findings that the mere presence of a gut microbiota reduces TT. However, we found no differences in TT between the two recipient groups. In the second experiment, we transplanted two groups of female GF C57Bl/6J mice with cecal material from two different conventional C57Bl/6J mouse donor groups treated with the TT-increasing drug loperamide or a saline vehicle. Again, no differences in TT were observed between the two recipient groups. These findings indicate that either the transferred microbiota did not engraft effectively, or that gut microbiota composition itself is not the principal driver of inter-individual TT variation.

## Introduction

Intestinal transit time (TT), which varies from around 12 h to more than 72 h in healthy individuals (Procházková et al., 2024), contributes to gut microbiome variation in humans (Vandeputte et al., 2016; Falony et al., 2016; Müller et al., 2019; Asnicar et al., 2021; Procházková et al., 2024). In observational studies, increased TT has been linked to higher alpha diversity (Roager et al., 2016; Vandeputte et al., 2016; Müller et al., 2019; Asnicar et al., 2021; Boekhorst et al., 2022; Procházková et al., 2024), increased proteolytic fermentation (Roager et al., 2016; Procházková et al., 2024), and increased abundance of specific microbial taxa, such as the mucin-degrading *Akkermansia* (Vandeputte et al., 2016; Falony et al., 2016; Asnicar et al., 2021; Procházková et al., 2024).

Although increasing evidence suggests that TT has direct effects on the gut microbiome, the relationship between the two is complicated by the fact that the link between them might be bidirectional. In a meta-analysis including 15 clinical studies, a multi-species probiotic decreased intestinal TT, increased stool frequency, and improved stool consistency in adults with functional constipation (Zhang et al., 2020). Similarly, fecal microbial transfer (FMT) from a healthy individual to patients with slow-transit constipation (STC) improved the symptoms of the FMT group compared to the control group receiving conventional treatment alone (Tian et al., 2017). The transfer of a long-transit phenotype from humans to mice has also been demonstrated. FMT from individuals with chronic constipation (Cao et al., 2017) or individuals with constipation-predominant irritable bowel syndrome (IBS-C) (Touw et al., 2017) into antibiotic-treated or germ-free (GF) mice increased the intestinal transit time of the animals when compared to mice transplanted with material from healthy donors. Multiple mechanisms by which the gut microbiota may impact intestinal TT have been suggested (Procházková et al., 2023). The microbially produced short-chain fatty acid (SCFA) butyrate has been demonstrated to increase the *ex vivo* contractile activity in the rat colon, potentially by increasing the proportion of excitatory myenteric neurons containing choline acetyltransferase (ChAT), producing the neurotransmitter acetylcholine (Soret et al., 2010). Serotonin is another important neurotransmitter in the gut that increases intestinal motility, and it has been demonstrated that gut microbiota-dependent serotonin signaling is essential for the maturation of the enteric nervous system (ENS) (De Vadder et al., 2018). Several microbial metabolites have been shown to stimulate intestinal serotonin release from enterochromaffin (EC) cells, including the secondary bile acid deoxycholic acid (DCA) (Alemi et al., 2013), SCFAs (Reigstad et al., 2015), and the tryptophan catabolite tryptamine (Takaki et al., 1985). In addition to microbial metabolites, the gases methane and hydrogen, which are both produced by intestinal microbes, can affect TT in opposite directions. In dogs, methane decreased TT *in vivo* (Pimentel et al., 2006) and reduced small intestinal contractility in guinea pigs *ex vivo* (Pimentel et al., 2006; Jahng et al., 2012), whereas hydrogen has been demonstrated to increase the colonic TT of guinea pigs (Jahng et al., 2012).

As described above, previous studies demonstrating the transfer of a long TT phenotype used feces from patients with either chronic constipation (Cao et al., 2017) or IBS-C (Touw et al., 2017). To our knowledge, no studies have investigated the transferability of TT from humans to mice with feces collected from healthy human donors with high inter-individual TT variation (Procházková et al., 2024). Thus, in the first part of this study, feces from healthy human donors with slow and fast colonic TT were used to colonize GF Swiss Webster mice, with subsequent assessment of TT in the recipient mice. An important limitation of human-to-mice transplantation studies is that only a subset of human gut microbiota-derived taxa colonizes successfully in the mouse gastrointestinal tract (Walter et al., 2020). Therefore, we additionally conducted a mouse-to-mouse cecum microbiota transplantation (CMT) study where groups of conventional C57BL/6J mice were first treated with the TT-increasing compound loperamide (or saline vehicle) for one week to increase intestinal TT, after which the animals were euthanized. Similar to a previous study (Touw et al., 2017), the cecal material was used to colonize groups of GF C57BL/6 mice, with subsequent assessment of TT.

## Materials and Methods

### Human donors

Fecal material used for transplantation was obtained from donors participating in a 9-day human study, conducted at the Department of Nutrition, Exercise and Sports (NEXS) at the University of Copenhagen (Denmark) from April to December 2021. The research protocol was approved by the Municipal Ethical Committee of the Capital Region of Denmark (H-20074067), and all participants provided written informed consent to participation. The study was registered at ClinicalTrails.gov (ID: NCT04804319), and a detailed study description has already been published (Procházková et al., 2024). In brief, all participants in the study reported being healthy (*i.e*., they did not suffer from inflammatory bowel syndrome, small-intestinal overgrowth, inflammatory bowel disease, chronic or infectious diseases, diabetes, or cancer) and had no medication use. Moreover, intake of antibiotics, laxatives, or diarrhea inhibitors 1 month before the trial was not allowed (Procházková et al., 2024). During the study, participants recorded their daily 24-h dietary records (over 8 days) and physical activity (over 9 days) (Table 1). Whole gut TT was estimated using a sweet-corn transit time test (Nestel et al., 2021) at two different time points, whereas segmental transit times for a subset of donors were assessed through SmartPill® ingestions followed by a standardized breakfast. Based on the transit times recorded during the SmartPill® ingestion (Table 1), two distinct donors with similar small intestinal transit, but varying colonic TT, were selected for the human-to-mice FMT study. Fecal material collected on the day of the SmartPill ingestion was used for the transplantation. Stool samples were collected by the donors, stored in their domestic freezers, and transported to the laboratory while being kept cold. After arriving at the laboratory, the fecal samples were stored at −20 °C overnight. In the morning, the samples were thawed and homogenized in sterile water 1:1, aliquoted to cryotubes, and stored at −80 °C (Procházková et al., 2024).

**Table 1:** Intestinal transit time of human donors. Small bowel transit time (SBT), and colonic transit time (CTT) for the two human donors, assessed through SmartPill® ingestion. Feces collected on the day of SmartPill® ingestion were used for FMT.

	Donor F (fast)	Donor S (slow)
Sex	Male	Female
BMI	27.6	21.9
Age (years)	28	27
Physical activity (min/day)	91.9	165
Medicine intake	No	No
Coffee (g/day)	574	398
Kcal per day	3,026	1,734
AOAC dietary fiber intake (g/day)	30	21
Englyst fiber (g/day)	5	6
Vegetables (g/day)	361	348
Fruit (g/day)	185	297
SmartPill SBT (hours)	4.2	4.5
SmartPill CTT (hours)	9.5	63.5
1^st^ corn WGT (hours)	30.4	62.5
2^nd^ corn WGT (hours)	13.6	41.7

### Animals

GF Swiss Webster mice (Tac:SW, originally obtained from Taconic Biosciences, Rensselaer, NY, USA) were bred and housed within GF isolators (Scanbur, Karlslunde, Denmark) at the National Food Institute, Technical University of Denmark. The mice were kept at a 12-h light cycle in a constant environment with a relative humidity of 55 ± 5% and a temperature of 22 ± 1 °C. The animals had *ad libitum* access to an irradiated regular chow diet (Altromin 1314; Brogaarden, Lynge, Denmark) and sterilized drinking water (Glostrup Hospital, Glostrup, Denmark). Before initiating the experiment, the GF condition of the mice was confirmed by plating fecal suspensions on blood agar plates (Statens Serum Institut, Copenhagen, Denmark), followed by both aerobic and anaerobic incubation.

Conventional C57Bl/6J mice (in-house breeding) were maintained at a 12-h light cycle in a temperature-controlled room (21 ± 1 °C) at Experimental Biomedicine, University of Gothenburg. The animals had *ad libitum* access to a regular chow diet (2019S; Inotiv, West Lafayette, IN, USA) and drinking water.

GF C57Bl/6J mice (in-house breeding) were maintained in flexible plastic gnotobiotic isolators at Experimental Biomedicine, University of Gothenburg. The mice were kept at a 12-h light cycle, a temperature of 20 ± 1 °C, and an air humidity of 45–70%. The animals had *ad libitum* access to an autoclaved regular chow diet (2019S; Inotiv, West Lafayette, IN, USA) and autoclaved drinking water. The GF status of the isolators was routinely monitored by anaerobic culturing and PCR amplification of the 16S rRNA gene.

Sample size estimations were based on a previous experiment that identified significant differences in TT between saline controls and loperamide-treated mice, using groups of six animals (Hjørne et al., 2025). However, for the conventional C57Bl/6J mice, only 11 animals were included due to limitations in litter size. All cages were enriched with autoclaved bedding material, wooden gnawing blocks, and red hides. However, during TT measurements, cages were covered with white paper, as described in the experimental procedures below, and only the hide was available. Prior to initiating the experiments, a list of humane endpoints was established for all animals involved in the experiments. These endpoints included markedly reduced food intake, bristly fur, behaviors indicative of pain, and a substantial weight loss (based on a visual estimate when a weight was not accessible in the isolators). The well-being of the animals was monitored daily. Researchers were aware of group allocations during both the assignment and statistical analysis phases. However, to ensure objectivity, the researcher conducting the TT measurements was always blinded to the grouping of the animals. To minimize confounding effects on TT measurements, all measurements were initiated in the morning, with measurements conducted simultaneously for animals within each experiment.

### Experimental design: human-to-mouse FMT

The human-to-mouse experiment was performed at the National Food Institute, Technical University of Denmark. The experiment was approved by the Danish Animal Experiment inspectorate (permit number 2020-15-0201-00484) and overseen by the in-house Animal Welfare Committee for Animal Care and Use. An unpublished internal study protocol outlining the experimental design, research question, and analysis plan was prepared prior to the initiation of the experiment. A total of 12 female GF Swiss Webster mice (8–12 weeks of age) were distributed into four isolators with three animals per isolator. The animals were randomized into the four isolators based on litter origin and body size (since weight measurements were not possible to obtain in the breeding isolators), ensuring an equal litter distribution and body size across groups. After 11 days of acclimatization, all animals were single-housed (Day 1). On Day 4, total intestinal TT was measured by administering 150 µL of an autoclaved solution of 6% carmine red (Sigma-Aldrich, St. Louis, MO, USA, C1022) and 0.05% methylcellulose (Sigma-Aldrich, St. Louis, MO, USA, M0262) through oral gavage. The animals were moved to clean cages with white paper covering the bottoms (still inside their respective isolators), and the time of gavage was noted for each animal, whereafter they were observed every 10–15 min until the first red pellet appeared. The TT was calculated as the time between oral carmine administration and the appearance of the first red pellet. After the first TT observation, the animals were divided into two groups: one group receiving fecal material from a human donor with a fast colonic transit time (Donor F) and one group receiving fecal material from a human donor with a slow colonic transit time (Donor S). For an overview of the intestinal transit time of the human donors, the reader is referred to Table 1. On the day following the first TT estimation (Day 5), the animals were transplanted with feces from their respective donors. The day before transplantation, the human fecal slurry was placed in the fridge (4 °C) to thaw overnight. The inoculum used for oral gavage was prepared in an anaerobic chamber by homogenizing the fecal slurry 1:5 in pre-reduced sterile phosphate-buffered saline (PBS) and centrifuging the homogenate at low speed (4 °C, 200 g, 3 min). The supernatants were immediately transferred to the animal facilities, and all animals were gavaged with 150 µL of fecal suspension from their respective donors. One animal was euthanized 4 days after the FMT due to substantial loss of body weight. Thus, the final number of animals was six in the Donor F group and five in the Donor S group. To assess potential acute and long-term effects of the FMT, total intestinal TT was measured two times after the FMT (4 and 24 days after FMT) as described above. Fresh fecal samples were collected in the morning on Day 31, and subsequently, the mice were reused for a dietary intervention reported previously (Sinha et al., 2024).

### Experimental design: mouse-to-mouse CMT

The mouse-to-mouse experiment was performed at Experimental Biomedicine, University of Gothenburg. The experiment was approved by the Research Animal Ethics Committee in Gothenburg, Sweden (Ethical approval number 4805-23). An unpublished internal study protocol outlining the experimental design, research question, and analysis plan was prepared prior to the initiation of the experiment. A total of 11 female conventional C57BL/6J mice (11–12 weeks of age) were included as donors, and 12 female GF C57BL/6J mice (13–14 weeks of age) were included as recipients. The conventional mice were housed 3–5 per cage in individually ventilated cages during the entire study period, except during the TT measurements as described below. The animals were divided into two groups: one group receiving a daily dose of 10 mg loperamide per kg body weight to increase intestinal transit time (Loperamide donors), and one group receiving a daily dose of sterile saline (Saline donors). Group allocations were based on litter origin, ensuring that animals from the same litter were distributed across different groups. A solution of 0.1% loperamide for oral gavage was prepared by dissolving pulverized Imolope® tablets from Orifarm Generics (2 mg of loperamide hydrochloride per tablet) in sterile saline as described previously (Hjørne et al., 2025). The animals were gavaged daily with loperamide/saline from Day 1 to Day 6, with volumes ranging from 0.155 to 0.210 mL. On the last day of treatment (Day 6), the TT of the animals was measured. On the morning of the measurement, all animals were moved to individual clean cages with white paper covering the bottom. TT was measured by administering an oral dose of 150 µL Evan’s blue solution (5% Evan’s blue in 1.5% methylcellulose) to all animals and recording the time between gavage and appearance of the first blue pellet. On the morning after the TT observation, all animals were anesthetized with isoflurane and euthanized by cervical dislocation (Day 7). The ceca from the three animals with the most extreme TT in each group were dissected, placed in a sterile petri dish, and transferred to an anaerobic container. Inside the anaerobic chamber, the ceca were opened and transferred to tubes containing pre-reduced sterile PBS. Ceca from the same donor group were pooled in a tube with 50 µL PBS. The ceca/PBS solutions were vortexed at maximum speed, glycerol was added to reach a final concentration of 6%, and the tubes were again vortexed at maximum speed. The solutions were allowed to sediment before the liquid was collected and aliquoted in sterile Hungate tubes, which were immediately transferred to −80 °C for storage.

On the day of colonization (Day 9), the cecal inocula were thawed at room temperature for 1 h and heated under running water just before the colonization. The GF mice were fasted for 4 h to ensure gastric emptying before they were gavaged with 150 µL of cecal inoculum from their respective donor group inside a sterile hood. Group allocations were based on litter origin, ensuring that animals from the same litter were distributed across different groups. Immediately after colonization, the animals were placed in sterile and individually ventilated isocages (ISOcage N system, Tecniplast, Buguggiate, Italy) with three animals per cage. However, due to problems with the isocage system, the animals were moved to regular individually ventilated cages after 4 days in the isocage system (Day 13). The cages were kept closed until the end of the experiment to avoid contamination. To assess the potential long-term effect of the CMT, the TT of the animals was estimated 13 days after colonization (Day 21), aligning with a previous study that assessed TT 15 days post colonization (Cao et al., 2017). All animals were moved to individual clean cages with white paper covering the bottom, and the animals were gavaged with 150 µL of a Carmine red solution (6% Carmine red in 0.05% methylcellulose). The time of gavage was noted, and the animals were observed every 10–15 min until the appearance of the first red pellet. After the TT observation, all animals were anesthetized with isoflurane and euthanized by cervical dislocation.

### DNA extraction and 16S rRNA gene amplicon sequencing

The Qiagen Powersoil DNeasy kit (12855-100; Qiagen, Hilden, Germany) was used to extract DNA from all human and mouse samples, as previously described (Laursen et al., 2021; Hjørne et al., 2025). DNA concentrations were measured using Qubit 2.0 dsDNA High Sensitivity (Qubit HS) kit (Q32851; Invitrogen, Waltham, MA, USA) and adjusted to 5 ng/μL for amplicon library preparation. PCR amplification and Ion Torrent GSS5 sequencing of the V3 region of the 16S rRNA gene were performed as previously described (Laursen et al., 2021; Sinha et al., 2024; Hjørne et al., 2025). Briefly, PCR was performed in a 20 µL reaction (17 μL Mastermix, 1 μL template DNA, and 2 μL forward primer, PBU 5′-A-adapter-TCAG-barcode-CCTACGGGAGGCAGCAG-3′, 10 pmol/μL). The primers were modified from Milani et al. (2013), and obtained from TAG Copenhagen A/S. The PCR was run on a 45-min program (30 s denaturation at 98 °C, 24 cycles of 98 °C for 15 s and 72 °C for 30 s, 5 min extension at 72 °C, and cooling to 4 °C), and the PCR products were purified with HighPrep PCR Magnetic Beads (AC-60005; MAGBIO, Gaithersburg, MD, USA) using a 96-well magnet stand (MAGBIO, Gaithersburg, MD, USA, MyMag 96), following the manufacturer’s instructions. Final DNA concentrations were measured using the Qubit HS kit (Invitrogen, Waltham, MA, USA, Q32851), and samples were pooled in equimolar concentrations before Ion Torrent sequencing. Sequencing was performed on a 318-chip using the Ion OneTouch™ 200 bp Template Kit v2 DL.

### Bioinformatic analysis

Raw 16S rRNA gene amplicon data were processed with an in-house pipeline (Mortensen, 2023a) as previously described (Hjørne et al., 2025). Briefly, demultiplexing was performed with cutadapt (v. 4.1) (Martin, 2011), denoising was performed with DADA2 (v. 1.22) (Callahan et al., 2016), and amplicon sequence variants (ASVs) were classified using the rdp_train_set_18 (Cole et al., 2014). Further processing and analysis of the data were done in R (v. 4.4.2). Sequencing data have been deposited at the NCBI Sequence Read Archive under the Bioproject PRJNA1044006 (human donors) and PRJNA1336477 (mice).

### Calculations & statistics

All statistical analyses were performed in R (v. 4.4.2), and all raw data and R scripts used for the analyses are available on data.dtu.dk (https://doi.org/10.11583/DTU.30146368). All *p*-values were adjusted for multiple comparisons using the False Discovery Rate (FDR) method. *P*-values below 0.05 after adjusting for multiple comparisons were considered significant. Appropriate parametric or non-parametric tests were applied for all analyses, depending on normality and variance tests. The tests applied for the specific comparisons are indicated in the figure legends. In all box plots, the border of the boxes indicates the interquartile range (IQR), horizontal lines represent the median, and the whiskers extend from the 25^th^ and 75^th^ percentile to the furthest outlier within 1.5 times the IQR. The dots represent individual data points, and group means are visualized with a cross. One animal from the human-mouse experiment was excluded from all statistical analyses due to euthanasia during the study, as detailed in the human-mouse section above. No other animals or data points were excluded from any of the statistical analyses. The primary outcome in both experiments was TT. Additionally, comparisons between donor and recipient microbiotas were conducted, as described below, to evaluate gut microbiota engraftment.

Before diversity analyses, rarefaction curves were generated, and based on these curves, each sample was rarefied to 20,000 reads to normalize the sequencing depth across samples. At this depth, all Shannon diversity curves had plateaued, while observed richness continued to increase slightly. However, the incremental gains were modest compared to the cost of excluding samples with lower sequencing depth. Alpha diversity indexes were calculated using the “calculate_alpha_diversity” function from the GMHmicrobiome package (Mortensen, 2023b) in R, and appropriate statistical tests were applied to test for differences between the groups (as indicated in the figure legends). For beta diversity analysis, we computed Jaccard distances (Jaccard, 1912), which quantify dissimilarity based on the presence or absence of taxa. Distances were calculated using the “distance” function from the phyloseq package (McMurdie & Holmes, 2013), and visualized through principal coordinates analysis (PCoA). Before testing for differences between groups, we calculated beta dispersion with the “betadisper” function from the vegan package (Oksanen et al., 2025) and applied a one-way ANOVA to identify possible differences between groups, which can affect the interpretation of the following PERMANOVA (Warton, Wright & Wang, 2012). Pairwise PERMANOVAs within the Ecole package (Smith, 2021) were performed for beta diversity analysis to test for differences between recipient groups. To assess whether each recipient group more closely resembled its respective donor than the other recipient group, we compared pairwise Jaccard distances between the two donors, the two recipient groups, and the donor-recipient pairs. To assess the efficiency of microbiota engraftment following FMT and CMT, we performed an ASV-level comparison between donor inocula and the corresponding microbiota profiles of recipient mice. For each donor-recipient pair, we identified ASVs present in the donor inoculum and assessed their presence in the corresponding recipients. Two metrics were calculated: (1) the proportion of donor ASVs detected in each recipient mouse (ASV retention), and (2) the proportion of each recipient’s total ASVs that were also found in the donor inoculum (proportion donor in recipient mouse).

## Results

### Transit time phenotype is not transferred by human-to-mouse FMT

**Figure 1:**
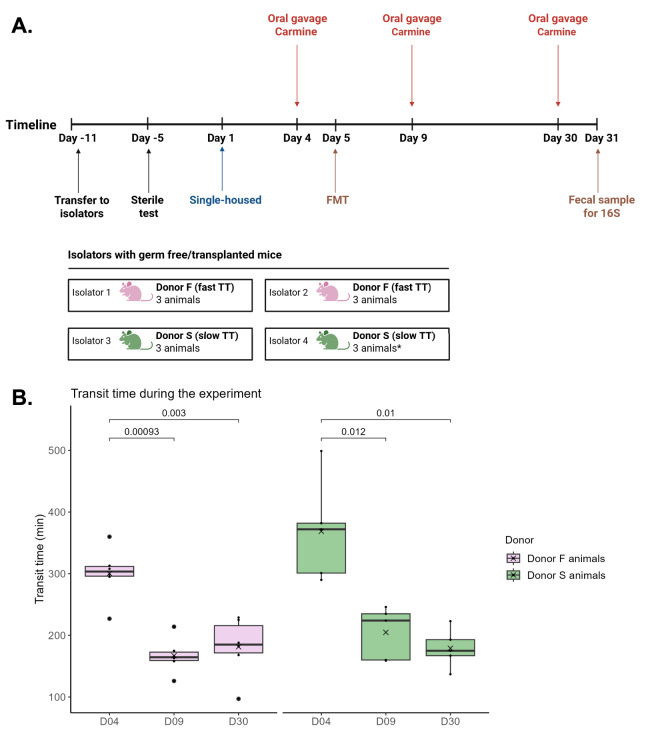
Study design and transit times for animals in the human-to-mouse FMT study. (A) Overview of the human-to-mouse FMT study design. OBS: One animal from isolator four was euthanized after the FMT due to substantial weight loss, meaning that isolator four only housed two animals. Created in BioRender. (B) Transit time (minutes) for the GF Swiss Webster animals before transplantation on Day 4, and after transplantation on Day 9 and Day 30. Differences between donor groups and between the different days were tested through a two-way ANOVA, which revealed a significant difference in TT between days. For each donor group, t-tests with FDR correction for multiple comparisons were performed to test significant differences between the days.

All animals were transplanted with fecal material from their respective donors on Day 5 of the study, and carmin-red TT estimations were performed on Day 4, Day 9, and Day 30 (Fig. 1A). A two-way ANOVA was conducted to assess differences in transit time between the two groups of animals and across the different days. No differences between the two groups of animals were observed at any of the time points (Fig. 1B). However, after the FMT, TT consistently decreased for both groups of animals (Day 4 *vs* Day 9 and 30).

### Microbial composition of human donor samples and mouse recipient feces

**Figure 2:**
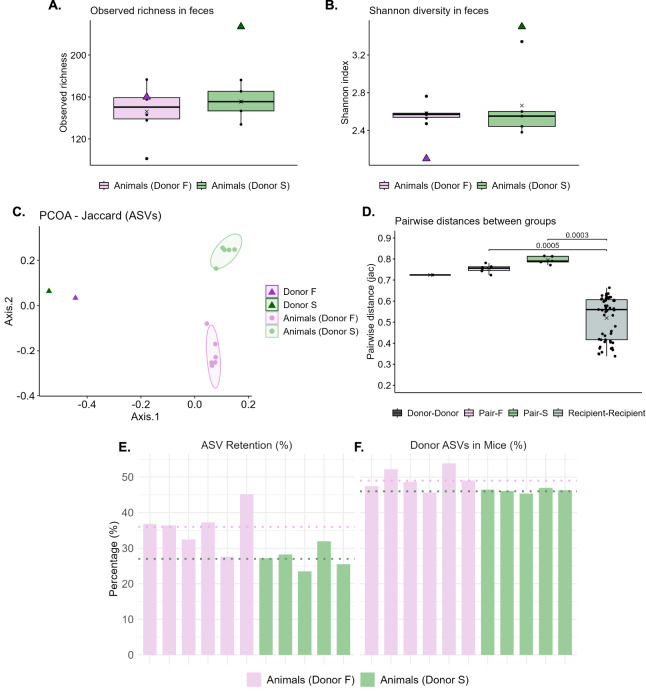
Microbiome description in the human-to-mouse FMT study. (A) The richness in the feces of recipient animals is illustrated with a box plot, whereas the richness in the feces of the human donors is illustrated with a triangle. An unpaired t-test was used to test for a difference between the recipient groups. (B) The Shannon index in the feces of recipient animals is illustrated with a box plot, whereas the Shannon index in the feces of the human donors is illustrated with a triangle. An unpaired Wilcoxon test was used to test for a difference between the recipient groups. (C) PCoA plot illustrating the Jaccard distance between the fecal microbiota of the recipient animals, as well as the fecal microbiota of their donors. Differences between the recipient animals were tested through a PERMANOVA. A significant difference was observed between Donor F (fast) and Donor S (slow) recipient animals (R^2^ = 0.39, *p* = 0.002). (D) The pairwise Jaccard distances between the two donors, the recipient animals and their donors, and the two groups of recipient animals. Differences between the groups of pairwise comparisons were tested through a Kruskal-Wallis test, followed by a Dunn’s test with FDR correction. (E) The percentages of donor-derived ASVs retained in recipient mice for each donor-recipient group. Each bar represents an individual animal, and dotted lines represent group means. (F) The percentages of donor ASVs within the total gut microbiota of the mice for each donor-recipient group. Each bar represents an individual animal, and dotted lines represent group means.

Gut microbial composition was assessed by 16S rRNA gene amplicon sequencing of DNA extracted from the human fecal samples and from the mouse fecal samples collected on Day 31 of the study (26 days after transplantation). A higher richness was observed in the feces of Donor S than in the recipient mice, whereas no difference in richness was observed between Donor F and recipient mice feces (Fig. 2A). Similarly, the Shannon diversity index was higher in Donor S feces compared to the recipient mice, whereas a lower index was observed in Donor F feces compared to the recipient mice (Fig. 2B). No significant differences in alpha diversity (Observed richness and Shannon index) were observed between the two mouse recipient groups (Figs. 2A, 2B). Beta diversity of the fecal microbial communities across groups was analyzed using the Jaccard distance at ASV level (Fig. 2C). A significant difference was observed between the two groups of recipient animals (R^2^ = 0.31, *p* = 0.002). The pairwise Jaccard distances between the donors, the recipient-donor pairs, and all recipients were compared. The pairwise distances between recipient-donor pairs were comparable to the distance observed between the two donors. In contrast, the pairwise distances among recipients were significantly smaller than their respective distances to donors, clearly indicating host selection of microbes (Fig. 2D). To evaluate the colonization efficiency of the human donor fecal microbiota in the recipient mice, we also quantified the percentage of donor-derived ASVs retained in the mouse gut microbiota and the proportion of donor-derived ASVs relative to the total ASV composition in the mice (Figs. 2D, 2E).

The mean ASV retention was 36% for the Donor F pair and 27% for the Donor S pair, indicating that a considerable proportion of donor-derived ASVs did not survive the FMT pretreatment or did not successfully engraft in the mice. The mean proportion of donor-derived ASVs within the total ASV composition of the recipient mice was 49% and 46% for Donor F and Donor S, respectively, indicating that a substantial fraction of the recipient microbiota may have originated from donor ASVs that were below the detection limit of 16S sequencing in the transplanted samples.

The relative abundance of the top genera across the donors and mice was visualized in a bar plot (Fig. S1). From a visual interpretation, the communities of the two recipient groups were more similar to each other than to their human donors, again indicating host selection of microbes. For the Donor F pair, *Bifidobacterium* and *Holdemanella* appeared markedly less abundant in the mice compared to the donor, while *Faecalibacterium*, *Bacteroides*, *Enterocloster*, and *Parabacteroides* appeared more abundant in the mice. For the Donor S pair, *Dialister*, *Flavonifractor, Mediterranea, Clostridium_IV*, and the family *Ruminococcaceae* appeared less abundant in the mice compared to the donor, while *Parabacteroides* and *Enterocloster* appeared more abundant in the mice.

### Transit time phenotype is not transferred through mouse-to-mouse CMT

**Figure 3:**
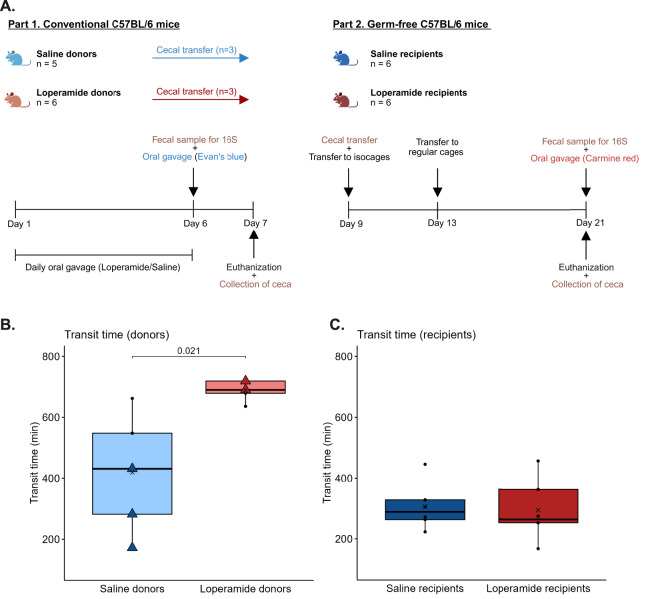
Study design and transit times for animals in the mouse-to-mouse CMT study. (A) Overview of the mouse-to-mouse CMT study design. Created in BioRender. (B) Transit time (minutes) for the conventional donor mice at the end of the treatment period. Mice used as donors are indicated with colored triangles. (C) Transit time (minutes) for the GF recipient mice 13 days after cecal transplantation. Differences between groups were tested through Wilcoxon tests followed by Dunn’s test for *post hoc* analysis.

To test whether TT could be transferred *via* mouse cecal material, conventional mice were treated for one week with either loperamide (10 mg/kg) or sterile saline. After treatment, TT was assessed, animals were euthanized, and cecal material was collected. In each group, the three mice with the most extreme TT values were selected as donors, and their cecal material was pooled and transplanted into germ-free recipients (designated Saline and Loperamide recipients; Fig. 3A). Thirteen days post-transplantation, a TT observation was performed, and the animals were euthanized. For the conventional animals, the TT of the loperamide-treated animals was significantly higher than the TT of the saline control animals at the end of the treatment period (Fig. 3B). However, for the recipient animals, no significant difference in TT was observed between the groups 13 days after transplantation (Fig. 3C).

### Microbial composition of donor mice and recipient mice

**Figure 4:**
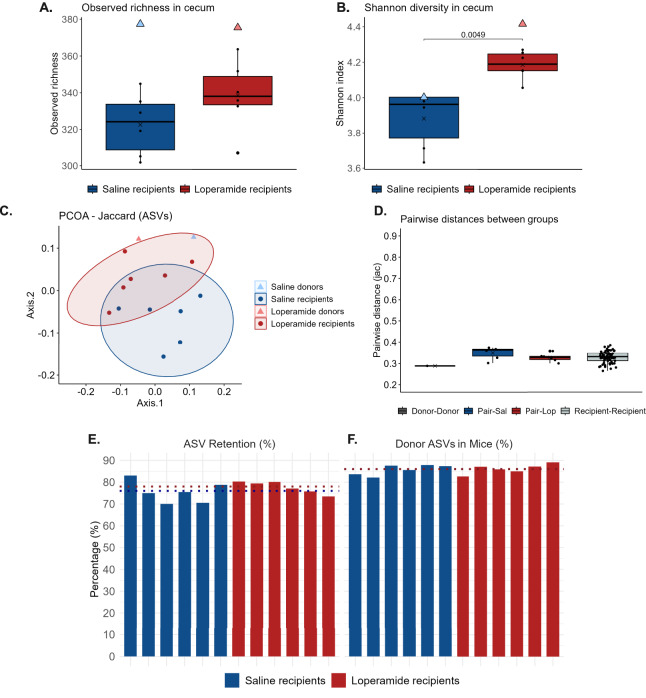
Microbiome description in the mouse-to-mouse CMT study. (A) The observed richness in the cecum of recipient animals is illustrated with a box plot, whereas the richness in the donor inocula is illustrated with a triangle. A t-test was used to test the difference between the recipient groups. (B) The Shannon index in the cecum of recipient animals is illustrated with a box plot, whereas the Shannon index for the donor inocula is illustrated with a triangle. A t-test was used to test the difference between the recipient groups. (C) PCOA plot illustrating the Jaccard distance between the cecal microbiotas of the recipient animals, as well as the communities of the cecal inocula. Differences between the recipient animals were tested through a PERMANOVA. A significant difference was observed between the loperamide and saline recipient animals (R^2^ = 0.13, *p* = 0.01). (D) The pairwise Jaccard distances between the two donors, the recipient animals and their donors, and the two groups of recipient animals. Differences between the groups of pairwise comparisons were tested through a one-way ANOVA (not significant). (E) The percentage of donor-derived ASVs retained in recipient mice for each donor-recipient group. Each bar represents an individual animal, and dotted lines represent group means. (F) The mean percentage of donor ASVs within the total gut microbiota of the mice for each donor-recipient group. Each bar represents an individual animal, and dotted lines represent group means (lines overlap due to identical group means).

To investigate the transfer of microbes from donor to recipient mice, 16S rRNA gene amplicon sequencing was performed on DNA extracted from the donor inoculum and from the ceca of recipient mice (collected during euthanization, 13 days after transplantation). A higher richness was observed in the inocula for both inoculum-recipient pairs (Fig. 4A). However, the Shannon index of the saline inoculum was similar to the Shannon index of the saline recipients, whereas a higher Shannon index was observed for the loperamide inoculum compared to the loperamide recipients (Fig. 4B). When comparing the two recipient groups, a significantly higher Shannon index was observed for the loperamide recipients (Fig. 4B). A similar tendency was observed for the observed richness (Fig. 4B), although not statistically significant. The beta diversity of the cecal microbial communities of the different groups was analyzed using the Jaccard distance (Fig. 4C). A significant difference was observed between the two recipient groups (R^2^ = 0.13, *p* = 0.03). For the pairwise Jaccard distances between donors, recipient-donor pairs, and among recipients, we found no significant differences across the comparisons, as well as short distances (0.29–0.35) compared to the distances in the human-mouse FMT study (0.52–0.80, Fig. 2D), indicating limited variation in overall community compositions (Fig. 4D). To evaluate the colonization efficiency of the donor cecal microbiota in the recipient mice, we also quantified the percentage of donor-derived ASVs retained in the recipient gut microbiota and the proportion of donor-derived ASVs relative to the total ASV composition in the recipients (Fig. 4E). The mean ASV retention was 76% for the saline donor/recipient pair and 78% for the loperamide donor/recipient pair, whereas the mean proportion of donor-derived ASVs within the total microbiota of recipients was 86% for both pairs, showing that most of the recipient microbial community originated from the donors.

The relative abundance of the top genera across the inocula and recipients was visualized in a bar plot (Fig. S2). From a visual interpretation, the cecal communities of the inocula and the recipient animals appeared very similar, again indicating a successful microbial engraftment. A slightly higher abundance of *Muribaculaceae* and *Paramuribaculum* seemed to be present in the saline recipients compared to the inoculum. For the Loperamide pair, a slightly higher abundance of *Paramuribaculum, Alistipes*, and *Oscilibacter* seemed to be present in the recipients, whereas a higher abundance of *Erysipelotrichaceae, Herbinix*, and *Bifidobacterium* seemed to be present in the inoculum.

## Discussion

Intestinal TT varies considerably between healthy individuals (Procházková et al., 2024) and several studies have suggested a direct effect of the gut microbiota on intestinal TT phenotype (Pimentel et al., 2006; Soret et al., 2010; Jahng et al., 2012; Alemi et al., 2013; Cao et al., 2017; Tian et al., 2017; Touw et al., 2017; Zhang et al., 2020; Li et al., 2021). Here, we were unable to transfer the TT phenotype to mice *via* FMT/CMT using either human or mouse donors. In the human-to-mice transplantation experiment, where GF mice received an FMT with human donor material, we observed a significant decrease in TT for both recipient groups after the FMT, as has also previously been reported (Kashyap et al., 2013; Touw et al., 2017). Thus, although we did not transfer the TT difference between the human donors to the mice, colonizing the GF animals with fecal bacteria did reduce the TT of both groups of animals. This suggests that the gut microbiota is important for gut function and TT, but may not be the primary driving factor for TT differences between healthy individuals. It should be mentioned that the experiments were conducted at different animal facilities, using distinct GF mouse strains as recipients. However, while this introduces additional variability, it can also be viewed as a strength, as reproducibility across facilities and recipient strains would enhance the robustness of potential findings.

Although previous studies have found a statistically significant effect on mouse TT after FMT from human donors (Cao et al., 2017; Touw et al., 2017) or mouse donors (Touw et al., 2017), the reported effect sizes in these studies are quite small. In one study, a mean increase in TT of approximately 20% was observed when comparing mice that received FMT from chronically constipated individuals to mice that received FMT from healthy controls (83.24 *vs* 69.06 min) (Cao et al., 2017). On the other hand, the defecation frequency of the constipated human donors was reduced by approximately 225% compared to the healthy donor group, with bowel movements occurring less than 2 times per week *vs* 6–7 times per week (Cao et al., 2017). In another study, a mean increase in TT of around 21% was observed for mice transplanted with cecal material from loperamide-treated animals compared to control animals (222 *vs* 183 min), although the TT of the loperamide donor group was increased by around 108% when compared to the control donor group (378 *vs* 182 min) (Touw et al., 2017). Thus, although transfers of the TT phenotype have previously been reported using both human and mouse fecal/cecal donors, the full effect has not been reproduced in any of the studies.

In addition to the possibility of the TT phenotype being untransferable, some limitations in our study may also explain the observed lack of phenotype transfer. In both experiments, the sample sizes included were smaller than in a previous study demonstrating a (partial) transfer of phenotype (16–17 animals) (Touw et al., 2017), and we cannot exclude the possibility that we would have detected a subtle effect if we had included a larger number of animals. However, our data does not suggest any tendencies in this direction, and in another study, five animals were sufficient to demonstrate a difference in TT following FMT (Cao et al., 2017). In the human-to-mice FMT study, we only included two human donors, and we cannot exclude the possibility that the phenotype could be transferred from other donors. Moreover, it was clear that a substantial part of the microbiota did not colonize well in the animals or did not survive pretreatment of the FMT material. As a result, taxa potentially important for the phenotype may not have been successfully transferred. Whether this aligns with other studies attempting to transfer TT is not clear, since these studies did not compare the microbiome composition of the human donors and mouse recipients (Cao et al., 2017; Touw et al., 2017). FMTs from humans to mice are complicated by the fact that important ecological factors of the host—such as diet, lifestyle, and genetics–are not co-transferred (Walter et al., 2020). Given that variables like diet, exercise, and age are known to contribute significantly to inter-individual variation in TT among healthy individuals (Procházková et al., 2023), this limitation could have a substantial impact on our experiment.

Although the TT phenotype was not transferred in the mouse-to-mouse experiment either, we transferred a substantial part of the gut microbiota from the donors to the recipients, underscoring the importance of host-microbiota compatibility in successful microbial engraftment. Moreover, we successfully transferred a higher Shannon diversity to the Loperamide recipient group compared to the Saline recipient group, mirroring the diversity observed in their respective donor groups. This diversity transfer is intriguing, given that higher fecal alpha diversity is consistently associated with longer TT in humans (Roager et al., 2016; Vandeputte et al., 2016; Müller et al., 2019; Asnicar et al., 2021; Boekhorst et al., 2022; Procházková et al., 2024). These findings suggest that microbiome features linked to long TT may not directly drive host motility, which depends on multiple host-specific and environmental factors, including pharmaceutical interventions such as the use of loperamide in this experiment. The effects of loperamide are well-described, and the compound increases transit time by decreasing peristalsis and fluid secretion in the intestine (Baker, 2007), with the effects on mouse TT being reversible after discontinuation of the treatment (Kashyap et al., 2013). Although loperamide-induced slowing of TT affect gut microbiome composition and activity (Kashyap et al., 2013; Wang et al., 2017b, 2017a; Hayeeawaema, Wichienchot & Khuituan, 2020; Li et al., 2020, 2022; Zhang et al., 2021; Tang et al., 2022; Hjørne et al., 2025; Kashyap et al., 2013; Wang et al., 2017b, 2017a; Hayeeawaema, Wichienchot & Khuituan, 2020; Li et al., 2020, 2022; Zhang et al., 2021; Tang et al., 2022; Hjørne et al., 2025), it is likely overly simplistic to assume that these microbiome changes alone can sustain prolonged TT in treatment-naïve animals, especially considering that microbiome features linked to drug-induced prolonged TT may represent adaptation rather than mediators that actively regulate motility. Moreover, an important limitation of using loperamide to prolong TT is that the drug may exert direct effects on the gut microbiome, potentially obscuring the distinction between drug-specific effects and those mediated by slowed TT. In a previous study (Hjørne et al., 2025), we observed that exposing fecal communities to loperamide *in vitro* affected the microbiome by reducing alpha diversity. However, taxa associated with drug-induced prolonged TT *in vivo* did not respond to direct drug exposure *in vitro*. Identifying a suitable mouse donor model for naturally prolonged TT may be challenging, as the natural variation in TT is limited in the inbred mouse strains commonly used in experimental studies. Finally, it should be noted that we used two different dyes to assess the transit time of the donor and recipient animals (Evan’s blue and Carmine red). From our experience, Carmine red is easier to detect in the mouse feces, but this dye was not available in the lab during the first measurement. The choice of dye could potentially influence transit time measurements, but to our knowledge, no published studies have directly compared these markers in an experimental setting. However, all groups that were directly compared within an experiment received the same dye, so this should not affect the interpretation of our results.

## Conclusion

In conclusion, the TT differences between donors were not transferred to GF recipient mice *via* FMT/CMT from either human or mouse donors in this study. It remains unclear whether the lack of phenotype transfer reflects insufficient colonization of key taxa or a lack of phenotype transferability. Our findings do not rule out a potential effect of the gut microbiota on host TT but indicate that the TT differences observed between healthy individuals are not universally transferable by FMT.

## Supplemental Information

10.7717/peerj.21064/supp-1 Supplemental Information 1 Relative abundance of fecal communities in the human-to-mouse FMT study. The relative abundance of the fecal microbiota on the genus level of Donor F (fast TT), Donor S (slow TT), Donor F mice, and Donor S mice. The most abundant genera are shown in the plot, while the remaining genera are grouped as “Other”.

10.7717/peerj.21064/supp-2 Supplemental Information 2 Relative abundance of cecal communities in the mouse-to-mouse CMT study. The relative abundance of the cecal microbiota on the genus level for Saline inoculum, Loperamide inoculum, Saline recipients, and Loperamide donors. The most abundant genera are shown in the plot, while the remaining genera are grouped as “Other”.

10.7717/peerj.21064/supp-3 Supplemental Information 3 ARRIVE Checklist.

## Additional Information and Declarations

### DNA Deposition

The following information was supplied regarding the deposition of DNA sequences:

Sequencing data are available at the NCBI Sequence Read Archive: PRJNA1044006 (human donors) and PRJNA1336477 (mice).

### Data Availability

The following information was supplied regarding data availability:

Sequencing data are available at the NCBI Sequence Read Archive: PRJNA1044006 (human donors) and PRJNA1336477 (mice).

Raw data and code are available at data.dtu.dk:

- Hjørne, Anna Pii (2026). Data for: Intestinal transit time phenotype is not transferred through gut microbiota transplantation. Technical University of Denmark. Dataset. https://doi.org/10.11583/DTU.30146368.v1.
