# Supplementary figures and images for: Intestinal transit time phenotype is not transferred through gut microbiota transplantation
Source: PeerJ. 2026 May 8;14:e21064. doi: 10.7717/peerj.21064 (PMC13159732; doi:10.7717/peerj.21064)

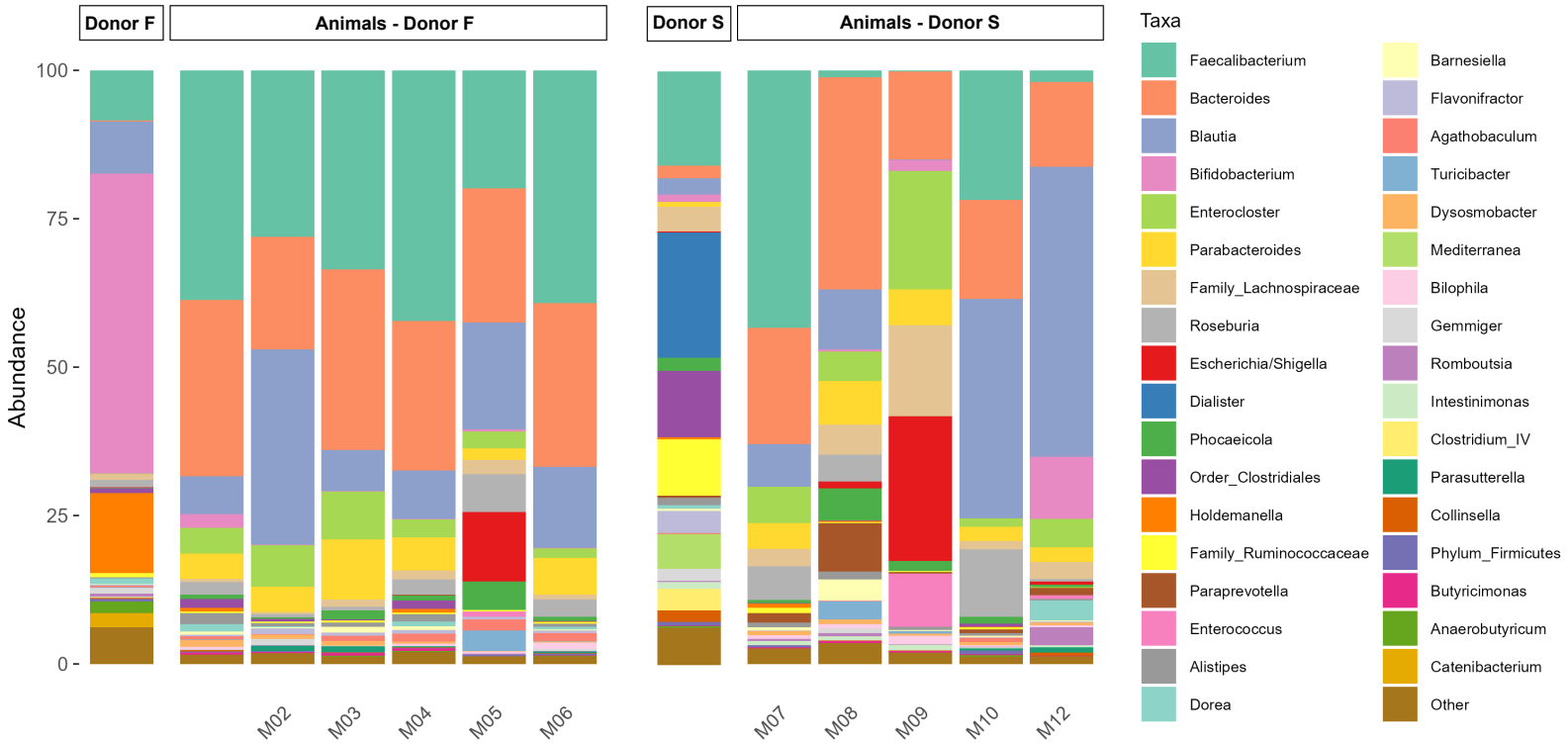

Supplement: Supplemental Information 1 — The relative abundance of the fecal microbiota on the genus level of Donor F (fast TT), Donor S (slow TT), Donor F mice, and Donor S mice. The most abundant genera are shown in the plot, while the remaining genera are grouped as “Other”. [file peerj-14-21064-s001.pdf]

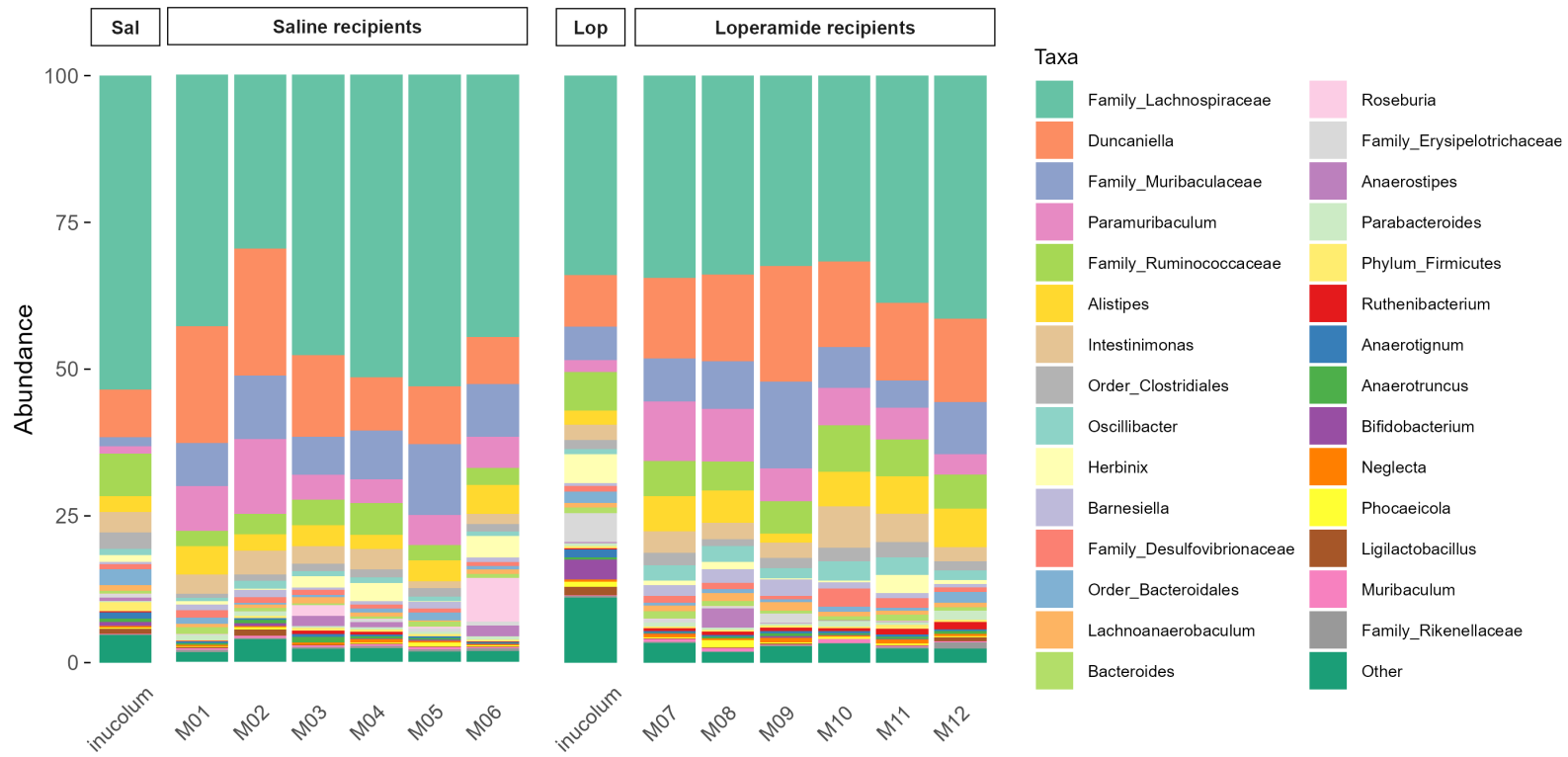

Supplement: Supplemental Information 2 — The relative abundance of the cecal microbiota on the genus level for Saline inoculum, Loperamide inoculum, Saline recipients, and Loperamide donors. The most abundant genera are shown in the plot, while the remaining genera are grouped as “Other”. [file peerj-14-21064-s002.pdf]
